# Early-Life Lung and Gut Microbiota Development and Respiratory Syncytial Virus Infection

**DOI:** 10.3389/fimmu.2022.877771

**Published:** 2022-04-04

**Authors:** Kazuma Yagi, Nobuhiro Asai, Gary B. Huffnagle, Nicholas W. Lukacs, Wendy Fonseca

**Affiliations:** ^1^Department of Pathology, University of Michigan, Ann Arbor, MI, United States; ^2^Department of Molecular, Cellular and Developmental Biology, University of Michigan, Ann Arbor, MI, United States; ^3^Mary H. Weiser Food Allergy Center, University of Michigan , Ann Arbor, MI, United States

**Keywords:** early-life microbiota, RSV, lung microbiota, gut microbiota, RSV-bronchiolitis

## Abstract

Several environmental factors can influence the development and establishment of the early-life microbiota. For example, exposure to different environmental factors from birth to childhood will shape the lung and gut microbiota and the development of the immune system, which will impact respiratory tract infection and widespread disease occurrence during infancy and later in life. Respiratory syncytial virus (RSV) infects most infants by the age of two and is the primary cause of bronchiolitis in children worldwide. Approximately a third of infants hospitalized with bronchiolitis develop asthma later in life. However, it is unclear what factors increase susceptibility to severe RSV-bronchiolitis and the subsequent asthma development. In recent years, the role of the gut and lung microbiota in airway diseases has received increased interest, and more studies have focused on this field. Different epidemiological studies and experimental animal models have associated early-life gut microbiota dysbiosis with an increased risk of lung disease later in life. This work will review published evidence that correlated environmental factors that affect the early-life microbiota composition and their role in developing severe RSV infection.

## Introduction

Respiratory Syncytial Virus (RSV) infection is the most prominent cause of bronchiolitis and childhood hospitalization in infants under six months of age (1, 2). Children under 2 years old are the most susceptible to developing severe RSV disease and asthma later in life. Interestingly, during the coronavirus infectious disease 2019 (COVID-19) pandemic, respiratory virus infection prevention approaches delay RSV season. However, after relaxing the control measures, several RSV outbreaks were observed in different countries that highlight the importance of this respiratory virus in children (3–5).

Infants hospitalized with RSV-bronchiolitis presented a higher risk of developing wheezing and asthma during their childhood. Prospective studies, using birth cohorts of infants, have determined that among 30 to 60% of infant experience RSV infection in the first year of life and by two years of age, almost all children will have been infected at least once (2, 6, 7). The main factors reported that increase the risk of developing severe RSV-bronchiolitis in healthy individuals are age (<6 months) and premature delivery (<35 gestational weeks) when the immune system is relatively immature (8). Although prematurity, has been identified as an independent risk factor for bronchiolitis (RSV and non-RSV bronchiolitis), the severity of the disease is worse in RSV infection (9). Even though all kids will be infected before 2 years of life, not all will develop severe RSV-bronchiolitis, suggesting that there may be other factors, intrinsic and extrinsic, that increased the risk of developing severe-RSV infection (10, 11).

After birth, the immune system of the infant is undeveloped and will evolve after recognizing environmental stimuli. The first contact happens during birth when the infants interact with commensal bacteria initiated upon the infant’s birth, vaginal track or skin, depending upon the mode of birth. The innate immune cells (neutrophils, macrophages, monocytes, and dendritic cells) develop and start maturation during the prenatal stage. Still, these cells have limited bactericidal function, suboptimal chemotaxis and responses to inflammatory stimuli, reduced cell adhesion and migration, and impaired innate signaling pathways that increase the risk of severe infection (12). Neonatal T cells are selected to become tolerant of self-antigens and reactive against foreign antigens. However, the activation of neonatal T cells generates a weak response to foreign antigens, and generally thought to be skewed towards Th2 immunity (12).

Different epidemiological studies and experimental animal models have associated early-life gut microbiota dysbiosis with disease risk later in life. The first years of life is a crucial time when the microbiota is established and when the immune system is maturing that represents a “window of opportunity” to prevent microbiome and immune alterations, such as asthma, allergies, severe respiratory infections, and autoimmune diseases (13–15). Exposure to different environmental factors from birth to childhood will shape the lung and gut microbiota and the development of the immune system, which may dictate the outcome of respiratory infections. The microbiome is involved in several essential processes, including metabolism and modulation of the immune system. The early-life microbiome plays a critical role in the infant’s overall health. The perturbations of the infant microbiome establishment and colonization can impact the long-term microbiota composition, potentially determining the predisposition to diseases during childhood and later in life (16). Infant microbiome composition is determined by the pre-and post-natal environmental exposures and will continue to evolve during the first years of life. Several factors affect microbiome composition in infants, including mother’s microbiome, mode of delivery (vaginal delivery or cesarean delivery [C-section]), smoke exposure, presence of pet in home, siblings, feeding options (breastmilk or formula), living setting (urban vs. farm), and the use of antibiotics during the first years of life (16–19). Some of these factors have been associated with increased predisposition for the development of immune-mediated diseases such as allergies and asthma (19). It has been reported that children living in rural communities develop a lower prevalence of allergies and asthma. The protection was associated with the increased diversity of microbial communities present in the house dust of these children compared with house dust of children living in non-rural communities (20). A similar effect was observed in children living with siblings or indoor/outdoor pets (21–23). Thus, the environment that the mother and child reside contributes to early immune development in health and disease.

The maternal influence on the development of the infant’s microbiome is the most compelling and related to choices in the pre-term and post-natal time frames. One of the most impactful factors that affect microbiome communities in infants is the mode of delivery (9, 18). Vaginal delivery exposes infants to the vaginal and intestinal microbiome of the mother and likely allows critical microbiome to become established early in the infant’s colon. For example, early vaginal bacteria from the birth canal include *Lactobacilli* that promote a desirable community to be established in the infant’s gut. In contrast, C-section exposes skin and environmental microbiome, such as *Staphylococcus*, generating distinct microbial acquisition that promotes a less desirable community. Thus, the mode of delivery impacts the establishment of the gut as well as lung microbiome and possibly the systemic and airway mucosal immunity. Elective C-section affects the gut microbiota up to 6 years of age. It has been reported that children born *via* C-section had an increased risk of diseases associated with the mucosal immune system, such as asthma, laryngitis, gastroenteritis, ulcerative colitis, lower respiratory tract infection (24). Wampch et al. reported similar microbial taxonomical succession trends between mothers (vaginal and feces samples) and infants (feces samples taken 1,3 and 5 days postpartum) in terms of diversity, evenness, and richness that highly correlated with metagenomic sequencing. Vaginal delivery infants presented a higher abundance of *Bacteroides* and *Parabacteroides* and lower *Staphylococcus* levels than C-section-born infants with a higher abundance of *Staphylococcus epidermidis* (25–27). In addition, the taxonomic difference in the gut of infants born *via* C-section vs. Vaginal delivery and the possible functional alteration using metagenomic sequencing data was examined. The overall changes observed were that the gut microbiota of the neonates born *via* vaginal delivery showed higher relative genes abundances in almost all pathways compared with C-sections. Vertical transmission of enteric microbial strains was studied by analyzing samples of mother-neonate pairs. Neonates born by vaginal delivery had multiple strains of Gram-positive bacteria (e.g., Bifidobacterium) transferred from their mother, while C-section delivered neonates had none (71% of vaginal delivery vs. 0% of C-section infants presented vertical transmission of this genus bacteria). Additionally, Gram-negative bacteria (e.g., Bacteroidetes) were transferred from mother to neonates in 79% of vaginal delivery neonates, and no vertical transmission was detected in C-section delivered infants (20–22). Interestingly, infants born *via* vaginal delivery had higher lipopolysaccharide (LPS) biosynthesis. This function was associated with Gram-negative bacteria transmitted from their mothers by vaginal delivery. Primary immune cells isolated from vaginally delivered infants produced higher tumor necrosis factor (TNF-α) and interleukin 18 (IL-18) when stimulated with LPS. This correlated with higher levels of these cytokines in blood plasma in infants born vaginally. This work demonstrated the critical role of the mode of delivery in the early-life gut microbiome establishment and its essential part in the maturation of the immune response.

Environmental exposure to microorganism during early life affect Toll-like receptors (TLR) expression. Microbial exposure increases the expression of *Tlr5* and *Tlr9* in newborn mice exposed (28); this correlates with the elevated expression of TLRs (*TLR2*, *TLR4*) in children born from mothers that live in environments with increased exposure to endotoxins (29, 30). Corroborating the importance of the mother’s microbiota and its transfer to newborns may impact the development of the immune system. Experiments on mice have shown that intranasal exposure to Gram-negative and Gram-positive bacterial polarized the immune response to Th1- cell-mediated immune response (31). This may help the neonates control better respiratory viral infection by maturation of their immune system.

In addition to the mode of delivery, studies have established that breastfeeding is considered a critical factor in shaping gut microbiota in early life. Breastmilk helps establish the early-life microbiome by providing viable bacteria in the milk transfer and further contributes essential prebiotics that appears to be provided exclusively by breastmilk to promote healthy gut microbiota colonization (17).

This manuscript will review the current literature to outline the critical role of the early-life lung and gut microbiome establishment and composition and their impact on the modulation of the immune response to RSV and other viral infections.

## Early-Life Respiratory Microbiome Composition in Health and Disease

Healthy lower airways have been formerly believed to be sterile or free from bacteria for a long time (32, 33). However, recent culture-independent molecular techniques for microbial identification such as pyrosequencing have elucidated that large numbers of microbial organisms, including bacteria, fungi, and viruses, collectively known as the respiratory microbiome, exist in the lower airway of healthy subjects and those with respiratory diseases (33, 34). Therefore, it has been considered that the composition of the respiratory microbiome is determined by the balance of three factors (35): (1) microbial immigration into the airways, (2) elimination of microbes from the airways, and (3) the relative reproduction rates of its community members found in the airways, which is determined by the regional growing conditions. Since the both acute and chronic respiratory disease can dramatically change these three ecological factors (35, 36), researchers have recently compared the respiratory microbiome during respiratory diseases with those of healthy subjects and have found significant differences in composition in patients comparing health and disease situations (37–40).

The upper respiratory tract has been recognized as the first line of defense, the main port of entry, and the reservoir for the respiratory microbiome (41, 42). Therefore, especially in newborns and infants, obligatory nasal breathers, it has been considered that microbiome composition of nasopharyngeal airways reflects exposures to the surrounding environment and shapes host immune responses, which will predispose to respiratory diseases later (33, 43–45).. With 16S ribosomal RNA (rRNA) pyrosequencing, Teo et al. reported that the nasopharyngeal microbiome in healthy infants could be divided into six relatively simple clusters based on the relative abundance of significant genera: *Moraxella*, *Haemophilus*, *Staphylococcus*, *Corynebacterium*, *Streptococcus*, and *Alloiococcus* (46). This latter study revealed that nasopharyngeal samples collected from healthy infants at around two months of age were dominated by *Staphylococcus* and *Corynebacterium* (46). Other longitudinal studies from European and American birth cohorts have shown that the nasopharyngeal microbiome of healthy infants presented a high abundance of *Corynebacteriaceae*, *Moraxellaceae*, and *Staphylococcaceae* in the first few months of life. After that, these microbes are subsequently replaced by the dominance of *Streptococcaceae* or either *Corynebacteriaceae* or *Moraxellaceae* and other minor bacterial families (45, 47–50). Although the active role of *Moraxellaceae* has not yet been consistent between studies, these microbial profiles in early life have also been shown to be related to protection against subsequent respiratory infections in later childhood (46, 47, 51).

The nasopharyngeal microbiota composition in children has been associated with diseases manifestation. A study by McCauley et al. analyzed nasopharynx swabs from kids with an upper respiratory infection and identified the dominant presence of *Moraxella* in the nasopharyngeal of children with upper respiratory tract infection in children. Suggesting the respiratory tract infection is associated with the presence of *Moraxella*-dominant nasopharyngeal microbiota (52). A different study investigating environmental drivers that alter respiratory microbiota composition that increased the risk of respiratory tract infection studied a prospectively followed cohort of infants. Nasopharyngeal microbiota samples of children from birth to 2 years old, with 11 consecutive samples, were characterized. The authors observed that infants with a higher predisposition of respiratory tract infection in their first year of life had an aberrant microbial developmental trajectory from the first month of life when compared with the group of infants with diminished airway infection. The altered nasopharyngeal microbiota presented decreased microbial community stability, reduction of *Corynebacterium* and *Dolosigranulum*, enrichment of *Moraxella* very early in life, followed by later enrichment of *Neisseria* and *Prevotella* spp. Interestingly the authors identify the mode of delivery, infant feeding selection, crowding, and antibiotic used as an independent driver of the altered respiratory microbiota (53). This work demonstrated the significance of the environmental factors that impact the respiratory tract microbiota and its association with the predisposition to respiratory tract infections.

### Early-Life Respiratory Microbiome and RSV Infection

Several epidemiologic studies have revealed that severe RSV infection is linked to the later development of hyper-reactive airway diseases such as recurrent wheezing and asthma (54–56). Severe RSV infection in early life could also interfere with the development of an appropriate host immune response and result in the structural and functional immaturity of the lung (57–59). Previous studies showed that a shift in the respiratory microbial composition during RSV infection might worsen the disease severity (60–62). A prospective study of 106 children aged two years or younger with the first episode of RSV infection revealed that hospitalization for RSV infection was positively associated with *Haemophilus influenzae* and *Streptococcus* and negatively with *Staphylococcus aureus* abundance (60). Moreover, the nasopharyngeal microbiome composition dominated by *Haemophilus influenzae* and *Streptococcus* was associated with more severe RSV infection and strengthened host immune response, characterized by overexpression of some genes such as interferon (IFN)-related genes, toll-like receptor genes, and genes linked to neutrophil recruitment and activation (60). A multicenter study examining infants hospitalized for bronchiolitis showed that *Haemophilus*-dominant nasopharyngeal microbiome profile was associated with a higher risk of intensive care, even after adjusting for confounders, including viral infection load (63). Ederveen et al. evaluated the nasopharyngeal microbiome compositions of infants younger than six months of age hospitalized for RSV infection. They revealed that transcriptome profiles enriched with *Haemophilus* were related to increasing interleukin 6 (IL-6) and C-X-C motif chemokine 8 (CXCL-8) responses in the nasopharyngeal aspirates (64). These findings suggest that respiratory early-life respiratory microbiome composition is associated with the severity of RSV infection and highlight the impact of the respiratory microbiome in the modulation of the immune response to respiratory tract infections

A comprehensive study by Raita et al. analyzed samples from infants hospitalized for bronchiolitis that were part of a multicenter prospective cohort study that involved 221 infants under one year of age were selected for nasopharyngeal microbiome analysis, transcriptome, and metabolic profiling (65). The authors identify a group of infants with a high risk of developing asthma later in life characterized by a high proportion of parental asthma and rhinovirus coinfection, with codominance of *S. pneumoniae*/*M. catarrhalis*, increased IFN-α and IFN-γ (65). Thus, there may be preferential opportunistic bacteria that are particularly associated with lung disease that can drive pathogenic sequelae.

In terms of the relationship between respiratory microbiome compositions and recurrent wheezing that occurs years after primary RSV infection, a study of infants aged six months or less hospitalized for severe RSV bronchiolitis reported that a higher relative abundance of *Haemophilus*, *Moraxella*, and *Klebsiella* was detected in infants who later developed recurrent wheezing as compared to in those who did not by the age of 3 years (66). This study showed a high abundance of *Haemophilus* and its association with an elevated level of CXCL-8, while *Moraxella* was associated with elevated IL-6 and IL-10 in the sputum supernatants (66). The multicenter study of infants conducted in the United States also reported that an increase in the relative abundance of *Moraxella* or *Streptococcus* species three weeks after bronchiolitis-related hospitalization was associated with an increased risk of recurrent wheezing by age three years (67). In addition, Hyde et al. reported an increase in *Haemophilus influenzae* and *Moraxella catarrhalis* discriminated between children with RSV/human rhinovirus (HRV) coinfection and those with RSV+ or HRV+ only (68). Interestingly *H. influenzae* was detected in most RSV+ infected samples and RSV/HRV-coinfected samples, not in samples from children infected with HRV+ only. As for *M. catarrhalis*, it was detected in coinfected samples, but not in samples from children RSV+ o HRV+ only (68). These results indicate that specific respiratory virus infections might promote the presence of specific bacterial species or vice versa in the context of bronchiolitis and predispose to subsequent development of recurrent wheezing or asthma in early childhood.

## Early-Life Gut Microbiome Composition in Health and Diseases

The gut microbiome composition in infants start to incorporate different microorganisms beginning at birth with some that persist as part of their long term gut microbiota. Although gut microbes have been identified in newborns shortly after birth, in vaginal delivery infants, their gut microbiome initially resembles their mother’s vaginal microbiota, containing mostly *Lactobacillus*, *Prevotella*, or *Sneathia*. In contrast, C-section delivery newborns present increased *Staphylococcus*, *Corynebacterium*, and *Propionibacterium*, which are common organisms found populating the skin (69). The infants will change the composition during the first year of life and increase diversity. The gut microbiome will start to resemble the adult microbiome around three years of age when it is mainly established (70, 71). The first bacterial communities found in infants’ gut are aerobic facultative or anaerobic bacteria (*Enterobacteria*, *Enterococcus*, and *Staphylococcus*). During their expansion, these groups of bacteria will consume oxygen. As a result, more anaerobic bacteria will increase, and the first anaerobes will emerge (*Bifidobacteria*, *Clostridia*, and *Bacteroides*). *Bifidobacteria* is one of the most predominant bacterial genera in human infants (72).

Breastfeeding is considered a factor that affects the gut microbiome and the immune response development during early -life. Human breastmilk contains 10^2^ to 10^4^ viable bacteria per mL, with the dominance of *Staphylococci*, *Streptococci*, *Lactobacilli*, and *Bifidobacteria*. Additionally, breast-skin microbiota is a source of microorganisms for the infant (73). It has been reported that breastmilk contains prebiotics, like oligosaccharides, which drive gut microbiome diversity in infants. *Lactobacillus*, *Staphylococcus*, *Enterococcus*, and *Bifidobacterium* are transferred to the infant during breastfeeding. In contrast, formula-fed babies’ gut microbiota is mainly integrated with facultative anaerobes, such as *Bacteroides* and *Clostridia*. The greater content and frequency of *Clostridia* in formula-fed infants is predominated of *Clostridium perfringens* (74). These differences in microbiome communities in breast vs. bottle fed infants correlate to the development of allergic diseases later in life, with breastfeeding being protective.

Early-life gut microbiome perturbation has been suggested as one of the most influential factors that impact human immune development (75). Accumulating evidence has highlighted that the gut microbiome influences lung immunity, although the mechanisms and underlying pathways are still under investigation. It has been reported that metabolites derived from bacterial fermentation of dietary fibers play an important role on local and systemic signaling molecules in sustaining immune and tissue homeostasis (76, 77). Gut microbiota alteration during early life leads to changes in the microbiome-derived metabolites profile of the infants, such as short-chain fatty acids (SCFAs) that are known to modulate the immune system and are implicated in the reduction of airway inflammatory infiltration. In addition, the changes of these metabolites have been associated with predisposition to food allergies (78–80) as well as having an important effect on epithelial barrier maintenance, which has a protective effect on the development of eczema early in life (81).

Immune-mediated diseases, like asthma, have both genetic and environmental components, but genetic factors cannot explain the increased incidence of asthma in the last 3 decades. It has been suggested that environmental factor exposures during early life that impact microbiota composition are associated with increased asthma prevalence (82). Arrieta et al. compared the gut microbiota of infants in the first years of life in a birth cohort of more than 300 kids and followed the infants from pregnancy until five years of age (19). In the latter study, the authors identify a link between gut microbiota dysbiosis in the first 100 days of life and an increased risk of developing asthma later in life. The gut dysbiosis was characterized by a reduction in four bacterial genera *Lachnospira*, *Veillonella*, *Faecalibacterium*, and *Rothia*. The authors confirmed this association using germ-free mice inoculated with stools from the asthmatic patient and supplemented mice with those four bacteria taxa and observed decreased lung inflammation after allergen challenge, suggesting that gut bacteria dysbiosis during infancy increased the risk of asthma later in life. In the same study, the authors investigated the metabolic profile of the infants in feces and urine. The authors observed a correlation in the presents of acetate in the feces of asthmatic kids, while the urine had altered secondary bile acids (glycolithocholate, glycocholenate, glycohyocholate, and tauroursodeoxycholate) (19). This work supported the importance of gut microbiome dysbiosis during early life and the predisposition of asthma and highlights the correlation in metabolites to disease that may influence the process.

One of the most common perturbations during early-life gut microbiome establishment is antibiotic therapy. Antibiotics in infants are commonly used and the most prescribed medication in preterm neonates in the neonatal intensive care units because of the high risk for infection (83, 84). Interestingly, antibiotic-induced dysbiosis in preterm infants has been associated with necrotizing enterocolitis (85). Analysis of stool samples from 84 longitudinal sampled preterm infants showed that the use of meropenem, cefotaxime, and ticarcillin-clavulanate were associated with significantly decreased species richness. The use of gentamicin and vancomycin has a variable effect on species richness (86). The effect of antibiotics used in children has been evaluated in a longitudinal study, where stool samples and clinical information were taken from 39 children. Around half of the children in the study received multiple courses of antibiotics during the first 3 years of life, and their gut microbiota was less diverse in terms of bacterial species and strains. Reduced microbiota diversity and its restoration after antibiotics are used take approximately one month (87). The study of early life antibiotic treatment and its effect on the gut microbiome and lung injury was evaluated using neonatal hyperoxic lung injury animal models. Mothers and offspring were treated with antibiotics before and after delivery, followed by 95% oxygen exposure. Microbiome analysis showed that perinatal antibiotics alter gut beta-diversity but not alpha diversity. In contrast, hyperoxia exposure in antibiotics-treated mice affected intestinal beta diversity and relative abundance of commensal bacteria. Interestingly, hyperoxia disrupted lung alveolarization and vascularization, but antibiotics used did not affect lung injury (88). Therefore, hypoxia and antibiotics generated gut microbiome dysbiosis but did not influence neonatal hyperoxic lung injury. Russell SL et al. showed that administration of vancomycin to neonatal mice altered the gut microbiome and increased the susceptibility to allergic asthma. This effect was not observed in mice treated with streptomycin. Interestingly, this effect was restricted to neonate mice, as adult mice did not show effects after antibiotic treatments. Early life administration of vancomycin, elevated serum IgE and reduced regulatory T cells in the lung (75). This work highlighted the importance of the early-life gut microbiome and the impact on lung immunity to allergic diseases.

These studies and others suggest the critical role of the early-life gut microbiome its impact on the maturation and programming of the immune system. Thus, the gut-lung axis is essential for maintaining homeostasis and training the host immune system and can contribute to long-term effects on health and disease.

### Early-Life Gut Microbiome and RSV Infection

While most infants infected with RSV develop mild disease, significant numbers of children will develop moderate to severe RSV disease that requires hospitalization, oxygen supplementation, or possibly intensive care. Harding et al. investigated stool samples collected by 95 babies, 37 healthy and 53 RSV-positive infants hospitalized (moderate RSV-disease) and 5 RSV-positive infants transferred to the pediatrician intensive care unit (ICU) (Severe RSV-disease) (89). All stool samples were collected within 72 hours of admission to analyze gut microbiota. The authors observed significant enrichment of *Clostridiales*, *Odoribacteraceae*, *Lactobacillaceae*, and *Actinomyces* in the RSV-positive infants’ (moderate and severe) samples compared with the healthy group. In addition, beta-diversity representing the overall microbial composition was significantly different between the RSV-positive group and the control group. In the infants with severe RSV-disease group, the authors observed slightly lower alpha diversity, representing the richness and evenness of the bacterial community compared with moderate RSV-disease and healthy controls. While this study has limitations since it cannot determine if the changes are the cause or the consequence of the infection, the timing of the sample collection (72 hours post-hospitalization) could indicate that early-life gut microbiome composition correlates with RSV-disease severity (89). Considering the hypothesis of the gut-lung axis, it is reasonable that gut dysbiosis affects disease severity in patients with RSV infection. Hasegawa et al. conducted a case-control study among hospitalized infants with bronchitis in investigating the correlation between fecal microbiome profile and bronchitis (90). Bronchiolitis for RSV infection was detected in 65% of the cases and rhinovirus in 23%. Furthermore, they demonstrated that, compared with infants with the *Enterobacter*/*Veillonella*-dominant profile, those with a *Bacteroides*-dominant profile had a higher likelihood of bronchiolitis (90). Thus, no matter the cause or effect relationship the differences in gut microbiome in RSV infected infants may play a longer-term role in subsequent disease processes due to the effects on the host immune responses.

As previously indicated, breastfeeding vs. formula feeding impacts the infant’s gut microbiome and overall immunity. In a retrospective study, medical records of more than 100 infants under one year of age that were hospitalized for RSV-bronchiolitis were studied. The authors observed infants that breastfed presented a less severe RSV-disease characterized by decreased need for oxygen therapy compared with formula fed babies, possibly correlating to decreased lung damage and lower risk of developing of asthma later in life (91). A similar study observed that hospitalized breastfed infants with RSV had reduced duration of hospitalization and lower rates of requiring oxygen therapy (92). While additional investigations are needed, these studies showed strong correlations of early-life alteration of the gut microbiome and the protection to the lung during RSV infection.

The critical impact of mode of delivery and the early-life gut and lung microbiota has been demonstrated, as indicated above (16, 53, 69). A national registry-based cohort study that included all the children born in Denmark during 1997-2003 followed hospitalizations for RSV in all infants from 0 to 23 months of age. The authors compared the effects of mode of delivery (C-section versus vaginal delivery) on the subsequent hospitalization for RSV infection (93). They observed that delivery by C-section was associated with an increased risk of hospitalization and severe-RSV disease, and this effect is persistent throughout the first two years of life (93). Several studies have indicated that C-section vs. natural birth alters the microbiome development in infants, suggesting a correlation with the above study further linking RSV infection severity with gut microbiome.

The role of the gut microbiome and microbiota-derived metabolites related to RSV infection has been studied in animal models by different groups (94–96). Gut microbiome modification in mice by supplementation with probiotics *(Lactobacillus johnsonii*) impacted the lung immunity to RSV experimental infection in mice (96). In this work, the authors observed that gut microbiome modification affected lung immunity by systemic metabolites, including docosahexaenoic (DHA) altered by the *L. johnosonii* supplementation. The metabolic reprogramming appeared to modify the phenotype of the dendritic cell precursors and led to attenuated immune responses to RSV (96). Subsequently, Fonseca et al. (94) demonstrated that maternal *L. johnsonii* supplementation impacted the offspring’s gut microbiome and was associated with RSV immune responses during neonatal infection that decreased airway mucus and Th2-mediated immune responses. Offspring from *L. johnsonii*-supplemented mothers had an expansion of bacteria belonging to *Lachnospiraceae* and *Muribaculaceae* genera similar to *Lactobacillus*-supplemented mothers. The metabolic profile of the plasma of mother and offspring as well as the mother’s breastmilk displayed similarity in the decreased presence of inflammatory metabolites (9,10-dihydroxyoctadecenoic acid (DiHOME), a linoleic acid metabolite, and guanosine) (94). This study emphasizes the importance of the mother’s microbiome and the transfer of immune-modulatory metabolites from mother to offspring, *via* gut microbiome transfer, possible through *in utero* metabolic signaling as well as metabolites in the breastmilk.

Similar to direct probiotic supplementation metabolite-mediated changes can be initiated utilizing high fiber diets that promote healthy gut microbiota and increase the production of microbiome-derived metabolites impacting the host’s immune response against RSV infection (95). The study demonstrated that a high-fiber diet protected mice against RSV infection by reducing the total numbers of inflammatory cells in the lung compared with control-diet mice, while a low fiber diet presented exacerbated RSV disease. The high-fiber diet mice showed altered gut microbiota composition, increasing the relative abundance of *Lachnospiraceae* spp (95). These data correlated with increased acetate in the feces of the high-fiber diet known to promote SCFA production. Furthermore, supplementation of the SCFA, acetate, to mice recapitulated the protection generated in high fiber diet suggesting a causal effect. The microbiome changes were associated with increased expression of type 1 IFN in lung epithelial cells, an innate cytokine known for anti-viral effects. Another similar study by Antunes et al. also investigated the effect of microbiota derived SCFA during RSV infection where they analyzed gut microbial profiles and observed a significant association between the family *Dysgonomonadaceae* and acetate in the stool. The severity of bronchiolitis was associated with specific bacterial profiles: length of hospitalization was positively associated with the family *Bacteriodaceae*. Furthermore, mice treated intranasally with acetate presented decreased viral load and increased expression of type 1 IFN (*Ifnb*) and retinoic acid-inducible gene I (*Rigi*) in the lung and was confirmed using *in vitro* analysis in A549 cells (97). Together, these studies highlight the importance of diet, gut microbiome, and microbiome-derived metabolites, especially SCFA, in controlling immune responses to RSV infection.

## Concluding Remarks

**Figure 1:**
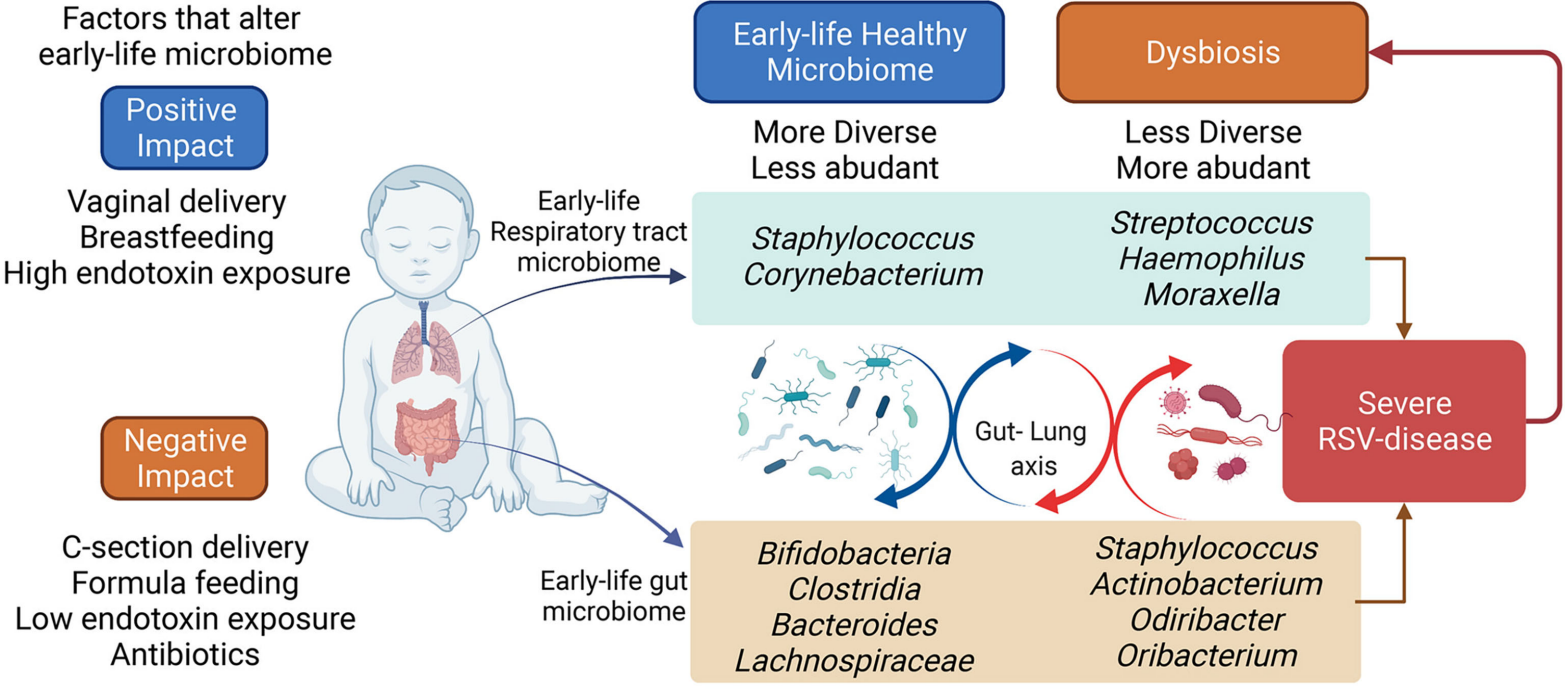
Early-life respiratory tract and the gut microbiome. The early-life microbiome plays an important role in the infant’s overall health. Several factors can affect the early-life microbiome establishment and colonization. The perturbations of the infant microbiome can impact the long-term microbiota composition, potentially determining the predisposition to inflammatory diseases, such as severe RSV disease. The bacterial composition in infants hospitalized for severe-RSV-disease is less diverse and more abundant, while healthy control displays a more diverse and less abundant bacterial composition. Severe RSV infection may alter the infant’s microbiome in the long term. Figure created with BioRender.com.

Epidemiological studies have linked pre-and post-natal exposures to be critical events for developing diseases during childhood. Of the many aspects related to these exposures, it has been shown that microbiome composition can be significantly altered and impact the development of the immune system by directly interacting with the cells or by promoting systemic mediators, such as metabolites. These early changes not only impact the early responses to infections and environmental allergens but also may have long-lasting effects on immune system function. The gut and lung microbiomes appear to promote immune maturation to control the development of inflammatory diseases, such as severe RSV that can predispose to asthma (**Table 1**) (13–15). Understanding the dynamics of the gut and lung microbiome during early life and how the alteration impacts the immune response of infants may allow the generation of interventional therapies, including prebiotics, probiotics and metabolites, that can help to shape the immune system’s development and activation (**Figure 1**). 

**Table 1:** Summary of early-life respiratory and gut microbiome alteration in infants.

Age	Site of microbiota, sample	Major outcomes	Reference
< 2 years	Respiratory, Nasopharyngeal swab	• Hospitalization for RSV infection was positively associated with *Haemophilus influenzae* and *Streptococcus* and negatively with *Staphylococcus aureus* abundance. • The nasopharyngeal microbiome composition dominated by *Haemophilus influenzae* and *Streptococcus* was associated with more severe RSV infection and strengthened host immune response.	(60)
< 1year	Respiratory, Nasopharyngeal aspirate	•* Haemophilu*s-dominant nasopharyngeal microbiome profile was associated with a higher risk of intensive care.	(63)
< 6 months	Respiratory, Nasopharyngeal aspirate	• Nasopharyngeal microbiome compositions enriched with *Haemophilus* were related to increasing interleukin (IL) IL -6 and C-X-C motif chemokine ligand -8 (CXCL-8) responses in the nasopharyngeal aspirates.	(64)
< 1 year	Respiratory, Nasopharyngeal aspirate	• Infants who are characterized by a high proportion of parental asthma and rhinovirus coinfection with codominance of *Streptococcus pneumoniae*/*Moraxella catarrhalis* had a high risk of developing asthma.	(65)
< 6 months	Respiratory, Nasotracheal aspiration	• A higher relative abundance of *Haemophilus*, *Moraxella*, and *Klebsiella* was detected in infants who later developed recurrent wheezing as compared to in those who did not by the age of 3 years. • A high abundance of *Hamophilu*s was associated with an elevated level of CXCL-8, and that of Moraxella was associated with elevated IL-6 and IL-10 in the sputum supernatants.	(66)
< 1year	Respiratory, Nasopharyngeal aspirate	• An increase in the relative abundance of *Moraxella* or *Streptococcus* species three weeks after bronchiolitis-related hospitalization was associated with an increased risk of recurrent wheezing by age three years.	(67)
< 2 years	Respiratory, Nasopharyngeal aspirate	•* Haemophilus influenzae* was detected in most RSV+ infected samples and RSV/Human rhinovirus (HRV)-coinfected samples, not in samples from children infected with HRV+ only. •* Moraxella catarrhalis* was detected in coinfected samples, but not in samples from children RSV+ or HRV+ only.	(68)
< 1 year	Gut, Stool	• Alpha diversity (the richness and evenness of the bacterial community) was slightly lower in the infants with severe RSV-disease group. • Beta diversity (the overall microbial composition) was significantly different between the RSV-positive group and the control group. • In the RSV-positive infants’ (moderate and severe) samples, significant enrichment of *Clostridiales*, *Odoribacteraceae*, *Lactobacillaceae*, and Actinomyces were observed.	(89)
< 1 year	Gut, Stool	• Infants with the *Bacteroides*-dominant profile had a higher likelihood of bronchiolitis as compared to those with the *Enterobacter*/*Veillonella*-dominant profile.	(90)

## Author Contributions

KY, NA, GH, NL, and WF conceived of and wrote the manuscript. All authors contributed to the article and approved the submitted version.

## Funding

This work was funded by National Institutes of Health grants R01HL138013 (NWL), RO1AI138348 (NWL, GBH), and R35HL150682 (NWL). WF was supported by a Parker B. Francis Foundation Fellowship.

## Conflict of Interest

The authors declare that the research was conducted in the absence of any commercial or financial relationships that could be construed as a potential conflict of interest.

## Publisher’s Note

All claims expressed in this article are solely those of the authors and do not necessarily represent those of their affiliated organizations, or those of the publisher, the editors and the reviewers. Any product that may be evaluated in this article, or claim that may be made by its manufacturer, is not guaranteed or endorsed by the publisher.
